# Effects of virtual reality simulation on medical students’ emotional and subjective experience compared to high-fidelity simulation in pediatrics clerkship

**DOI:** 10.1371/journal.pone.0323150

**Published:** 2025-10-08

**Authors:** Sejin Kim, Jang Hoon Lee, Hyun Joo Jung, Mira Kim, Su Kyung Lee, Jihye Yu

**Affiliations:** 1 Department of Medical Education, Ajou University School of Medicine, Suwon, Republic of Korea; 2 Department of Medical Education, Seoul National University College of Medicine, Seoul, Republic of Korea; 3 Department of Human System Medicine, College of Medicine, Seoul National University, Seoul, Korea; 4 Department of Pediatrics, Ajou University School of Medicine, Suwon, Republic of Korea; 5 Office of Medical Education, Ajou University School of Medicine, Suwon, Republic of Korea; 6 Ajou Center for Clinical Excellence, Ajou University School of Medicine, Suwon, Republic of Korea; Ankara University Faculty of Medicine: Ankara Universitesi Tip Fakultesi, TÜRKIYE

## Abstract

This study aimed to explore the educational impact of virtual reality (VR) simulation compared to high-fidelity (HF) simulation. Recently, VR simulation has been integrated into medical education, offering advantages such as cost- and space-efficiency, accessibility, and autonomy, enabling self-directed, repeated practice. Prior research comparing VR and HF simulations has focused primarily on knowledge acquisition, skill development, and learner satisfaction. Given that medical students often experience low confidence and high anxiety during clinical transitions, it is essential to examine the VR simulation’s impact on these emotional factors and students’ subjective experiences. This study investigated the effects of VR and HF simulations on anxiety, confidence, engagement, and perceived learning using multiple-item self-report scales. Data were analyzed using independent t-tests to compare the two simulation methods. The study included 40 fifth-year Korean medical students in a pediatric clerkship. Participants completed both VR and HF simulations, followed by a questionnaire assessing these variables. Results showed no statistically significant differences between VR and HF simulations across the measured domains. However, item-level analysis indicated slightly higher anxiety in VR simulation but greater perceived learning in cognitive domain, suggesting that VR may be more beneficial for cognitive learning compared to HF simulation. As VR simulation has only recently been incorporated into medical education, this study represents a first attempt to compare its impact with that of HF simulation focusing on subjective outcomes. Although no significant differences were found, the cost and space efficiency of VR simulation along with its support for autonomous learning underscore the need for further research, particularly on students’ experiences, and learning objectives.

## Introduction

Simulation-based education is a widely used teaching method in medical education [1]. It offers a controlled environment that replicates authentic clinical scenarios, yielding positive outcomes in knowledge acquisition and communication skill development [2]. Furthermore, it enables students to practice medical procedures without the stress and pressure associated with real-life situations [3]. Among the available methods, high-fidelity (HF) simulation, which uses mannequins as virtual patients to recreate a realistic medical setting, is widely integrated into the regular curriculum of medical education, yielding ample evidence that simulation aids healthcare students’ learning process. HF simulation enhances clinical competencies, including knowledge, skills, and decision-making [1,4–6], as well as nonclinical competencies, such as communication and collaboration skills, leadership, and critical thinking [1,4,7,8]. In addition, it fosters self-satisfaction and self-confidence [1,4,6,9,10]. However, HF simulations are not always feasible or accessible to schools and students because they require substantial financial, spatial, and human resources. To implement an HF simulation, a high-cost mannequin with an accommodating room is a prerequisite, along with an extra control room and trained personnel who can control the mannequins and provide feedback to students.

Meanwhile, virtual reality (VR) simulation has emerged as a transformative simulation method in medical education. VR simulations enable learners to experience environments typically inaccessible in real life [11,12] by transporting them into interactive virtual environments, which positively influence student learning [13]. VR simulation requires a head-mounted display (HMD), handheld controllers, a high-functioning computer or laptop, and a small physical space of approximately 4m^2^ [13]. Compared to the characteristics of HF simulation (see [3]), VR simulation offers comparable or even greater advantages in terms of low cost, realistic healthcare experience, the ability to pause and repeat practice, and support for both individual and group evaluations, all without patient safety concerns [13].

As VR simulations have begun to appear in medical education curricula, research on their effectiveness is expanding. However, a limited number of studies have compared VR simulations with other types of simulations and reported inconsistent results (for a review, see [14]). Some studies have reported that VR simulations outperform traditional simulations in terms of enhancing diagnostic accuracy and facilitating knowledge acquisition [15,16]. In contrast, other studies have found no significant difference in skill performance between VR and other simulation methods [17,18].

A handful of prior studies suggest that VR simulation is at least non-inferior to HF simulation in terms of knowledge acquisition and skill attainment. However, affective domains such as anxiety, confidence, engagement, and perceived learning remain largely unexplored despite that simulation-based learning can be effective in helping learners manage emotional responses [19]. One of the key challenges students face during internships is high anxiety and a lack of confidence in practicing their skills and meeting clinical demands [20]. High levels of anxiety can impair decision-making and negatively impact skill performance [21,22]. Similarly, a lack of confidence may lead to hesitation, impacting decision-making and clinical performance [23]. Minter et al. [20] proposed that preparatory programs, such as simulation-based training can address medical students’ lack of confidence and high anxiety, making them better prepared for clinical practice.

A growing body of evidence shows that HF simulation significantly reduces medical students’ anxiety and increases their confidence in clinical practice [24–26]. Likewise, VR simulation provides a psychologically safe environment in which students can practice and refine their skills without fear of judgment or error [27]; thus, it can be an alternative method for effectively handling affective variables [28]. In this context, whether HF or VR can better prepare students for emotional and attitudinal demands needs to be investigated.

Additionally, prior studies on the subjective experience of VR and HF simulations primarily focused on surface-level factors, such as student preferences or satisfaction (e.g., [29,30]) or the usability of VR simulators (e.g., [15,17]), showing that VR simulation is a relatively new educational method utilizing advanced technology. Although these approaches capture subjective learning experiences, they fail to address such experiences comprehensively. Although HF and VR simulations are known to induce high levels of student engagement by providing immersive settings and realistic clinical scenarios, no empirical studies have directly compared the engagement levels between these modalities. Furthermore, beyond assessing learner preferences or satisfaction with VR simulations, it is important to explore how learners perceive their learning within the simulation. Therefore, to comprehensively investigate and understand the educational effects of the two simulation methods in terms of subjective experience, levels of engagement and perceived learning must be systematically compared.

## Materials and methods

### Study design

This study was conducted in 2023 as part of a regular clinical clerkship program in pediatrics. During the clerkship, groups of eight to nine students participated in each rotation, with two to three students in each group engaging in both VR and HF simulations.

### Participants

43 fifth-year medical students from the Ajou University School of Medicine, Korea participated in this study. Since the simulation was a part of the formal curriculum, all fifth-year students enrolled participated in the study, representing the maximum number of participants possible within the educational context. Of these, two were excluded from the analysis because they showed a pattern of careless responding, consistently selecting the same option across items. Thus, 41 participants remained for the final analysis, including 29 males. This was their second experience with VR simulation, as they had a previous VR simulation experience in a pulmonologist clerkship before a pediatrics clerkship. As this study was a retrospective analysis using survey data gathered from the students, informed consent was exempted.

### Ethical considerations

This study was approved by the Institutional Review Board (IRB) of Ajou University Hospital (No. AJOUIRB-DB-2024–002). Ethical approval was obtained retrospectively from the IRB, as the data were collected after the completion of the regular curriculum. The IRB confirmed that informed consent could be waived, as the study posed minimal risk to the participants’ safety, rights, and well-being. The data used for research were accessed on February 6, 2024. Although individually identifiable information was included during the data collection phase, all data were anonymized before being used for analysis.

### Procedure

From May to November 2023, groups of fifth-year medical students participated in a four-week pediatric clerkship program. In the 3rd week, they engaged in a VR simulation, followed by HF simulation in the 4th week. Each simulation consisted of three stages: introduction – simulation – debriefing. In the VR simulation, students practiced using the controllers during the introduction stage. After being introduced to the case scenario, students participated in the simulation for about 15 minutes. The debriefing was part of the program and was led by a pediatrics faculty member. Right after the debriefing, students completed a questionnaire designed to assess anxiety, confidence, engagement, and perceived learning.

### Simulation setting and procedure

Fifth-year medical students participated in groups of 8–9 per clerkship rotation, with subgroups of 2–3 students engaging in each simulation run. The VR simulation was conducted in week 3 and the HF simulation in week 4 of the clerkship. All sessions were supervised by a pediatric professor for educational oversight and by an operator for technical support. Both simulations addressed the Neonatal Resuscitation Program (NRP), with the learning objective of “knowing and following the NRP algorithm.” As the students had already studied warning signs and NRP procedures, the simulations were designed to assess their ability to apply this knowledge and make clinical judgments in accordance with the protocol, therefore, no additional signals or guidance were provided during the simulation. The same professor supervised both simulations and evaluated performance using rubric.

The VR simulation utilized the case “Precipitous Labor & NRP,” developed by Sim-X. After the initial maternal assessment, the scenario progressed to neonatal resuscitation, which included four stages: initial assessment, positive pressure ventilation (PPV), cardiac arrest, and return of spontaneous circulation (ROSC). During the simulation, students were required to assess the situation, make clinical decisions based on the NRP algorithm, and perform the actions through the VR interface, communicating with their peers to coordinate care. The operator was able to manually register the students’ actions when technical issues with the controllers prevented automatic registration. The simulation was conducted using the MetaQuest 2 head-mounted display and controllers. Each subgroup was introduced to the case, practiced with the controllers for about 20 minutes, and then engaged in the scenario for approximately 15 minutes. Following the VR session, a pediatric professor conducted a debriefing based on the rubric.

The HF simulation employed Laerdal’s high-fidelity mannequin simulators, specifically SimMom and Premature Anne. The scenario progression was identical to that of the VR simulation, but the operator controlled changes in the vital signs of the maternal and neonatal simulators. In the HF simulation, students conducted physical assessments on the mannequin, initiated resuscitation steps according to the NRP protocol, and communicated with their peers to coordinate care. The same groups of students participated in the HF simulation for about 15 minutes, and each session was recorded. After each group completed the HF simulation, the same faculty member reviewed the recorded video providing rubric-based feedback.

### Measures

Anxiety, confidence, engagement, and perceived learning were assessed using the following scales: Anxiety was measured using the short form of the State-Trait Anxiety Inventory (STAI; [31]), which comprises six items. The scale was originally rated on a 4-point Likert scale (1 = Almost Never to 4 = Almost Always), but was modified to a 5-point Likert scale (1 = Totally Disagree to 5 = Totally Agree) for this study. The modification was made to ensure consistency across the instruments used in this study, which all employed a 5-point scale, and to prevent unnecessary confusion for respondents. Positive items such as “I am calm” were reverse coded. Higher scores indicated greater levels of anxiety. The scale demonstrated good internal consistency, with a Cronbach’s α coefficient of.86.

The Cato Confidence Scale [32] was adopted to assess confidence. Although the original scale consisted of 19 items, only 10 items specifically addressing confidence in the simulation and relevant to the simulation setting were included with modifications. For example, the item “When making a decision about the patient I feel…” was changed to “when working with the mannequin, I feel…” for the HF simulation, and “when working with the virtual patient, I feel…” for the VR simulation questionnaire. Each item was rated on a 5-point Likert scale ranging from “Very Anxious” to “Very Confident,” with higher scores indicating greater confidence. The scale demonstrated good internal consistency (Cronbach’s α = .87). Engagement was measured using the Flow in Education Scale [33], which comprises four domains: cognitive control, time transformation, loss of self-consciousness, and autotelic experience. Each domain was assessed using three items, resulting in 12 items. Responses were rated on a 5-point Likert scale ranging from 1 (Not at All) to 5 (Very Much). The internal consistency of Engagement was excellent, as indicated by Cronbach’s α = .93. Perceived learning was evaluated using the Perceived Learning Scale [34], with three items assessing learning in the cognitive, affective, and psychomotor domains. The item “My attitudes toward the subject matter in this course were more positive than before taking the course” was excluded to improve internal consistency. The remaining 16 items demonstrated a Cronbach’s α coefficient of.75, which is an acceptable level of internal consistency. The original 7-point Likert scale (1 = Strongly Disagree ~ 7 = Strongly Agree) was modified to a 5-point Likert scale for this study. The scales used in this study were used in prior studies on simulation in medical and nursing education (e.g., [35,36]). The complete set of survey questions is available in S1 File.

### Statistical analysis

SPSS version 25 was used for statistical analysis. Descriptive statistics were used to calculate the mean and standard deviation for each variable, and dependent t-tests were conducted to analyze the mean differences in the data. A significant level of.05 was used for all statistical tests.

## Results

Table 1 presents the levels of anxiety, confidence, engagement, and perceived learning reported by participants after each simulation. The findings suggest no statistically significant differences between the VR and HF simulations across the measured variables, on average.

**Table 1:** Comparison of anxiety, confidence, engagement, and perceived learning in VR and HF simulations between VR and HF simulations.

	VR simulation	HF simulation	*t*	*p*-value
Anxiety	3.26(±0.65)	3.22(±0.60)	0.41	.69
Confidence	3.15(±0.59)	3.15(±0.57)	0.14	.89
Engagement	3.53(±0.58)	3.53(±0.50)	−0.09	.93
Perceived learning	3.70(±0.42)	3.68(±0.33)	1.00	.33

A detailed analysis of individual items revealed significant differences in one item per scale, except for confidence. Table 2 lists the specific items for which statistical differences were found between the VR and HF simulations. On the Anxiety scale, participants reported significantly higher levels of anxiety for the item “I feel upset” during the VR simulation compared to the HF simulation, *t*(40) = 2.06, *p* = .046. Additionally, two items belonging to engagement and perceived learning showed higher scores in the VR simulation than in the HF simulation: *t*(40) = 2.22, *p* = .032, and *t*(40) = 2.30, *p* = .027, respectively.

**Table 2:** Items showing statistically significant differences in anxiety, engagement, and perceived learning between VR and HF simulations.

Scale	Item	VR simulation	HF simulation	*t*	*p*-value
Anxiety	I feel upset.	3.37(±1.00)	3.00(±0.95)	2.06	.046
Engagement	At each step, I know exactly what I have to do.	3.78(±0.65)	3.51(±0.68)	2.22	.032
Perceived learning	I can organize course material into a logical structure.	3.85(±0.65)	3.56(±0.78)	2.30	.027

## Discussion

This study compared the anxiety, confidence, engagement, and perceived learning outcomes for VR and HF simulations. The results showed that the two simulations did not produce significantly different levels of anxiety, confidence, engagement, or perceived learning. The absence of differences does not directly support that VR simulation is equally effective as HF simulation in maintaining students’ affective factors. This study may have failed to capture these differences due to various factors, such as an insufficient sample size to detect the impacts, inadequate measurement tools for assessing both VR and HF simulation experiences. Although our final sample size (*n* = 41) exceeded the threshold suggested by a conventional power analysis (*α* = .05, power = .80, medium effect size d = 0.5; [37]), it is possible that the true effect size between the two modalities was much smaller than anticipated, making it difficult to detect significant differences. Nevertheless, considering previous studies that compared VR and HF simulations, the lack of differences between the two simulations can be regarded as a consistent finding.

Prior studies in nursing education found non-inferiority of VR simulation to HF simulation in building self-confidence (for a review, see [38]), which supports the finding of this study. However, the findings in medical education are not consistent. Abulfaraj et al. [17] reported no significant difference in confidence levels between VR and HF simulations for managing patients with status epilepticus. But Macnamara et al. [39] found higher confidence in a HF simulation than in a VR simulation when performing airway, breathing, circulation, disability, and exposure (ABCDE) assessments. Both study applied a single item scale with 5-point likert scale to measure confidence. This study was the first to use a multi-item scale to measure psychological variables in comparing impacts of two simulation methods, further research is required to replicate the effects of the simulations on both anxiety and confidence.

An item-level comparison of the anxiety scale revealed that the students reported higher levels of discomfort during the VR simulation than during the HF simulation. Despite prior experience and familiarity with VR instruction, students could not always control their movements as intended in the VR environment, which may have contributed to their feeling upset. This finding aligns with that of a prior study suggesting that increased anxiety in VR simulations may stem from technological challenges rather than the simulation [40]. In the prior studies, participants reported that HF simulation is not easy to control as well [17,39]. Insufficient experience with VR simulators may contribute to discomfort, considering the finding of Palmisano and Constable [41] that cybersickness is reduced with repeated use of a head-mounted display.

This study also found no significant differences in engagement and perceived learning between HF and VR simulations. To our knowledge, this is the first study to compare student engagement and perceived learning across these two simulation modalities. A prior study similarly reported no significant difference in immersion between VR and HF simulations [39]. Given that ‘immersion and time transformation’ is a subscale of engagement, this finding suggests that engagement levels may not differ significantly between the two modalities. Moreover, as previous research has shown that confidence levels correlate with perceived learning in VR simulations [41,42], the lack of differences in confidence levels between the two modalities may explain the absence of perceived learning differences.

However, the sequential design raises the possibility of a confounding effect, in which prior experience with the VR simulation may have influenced students’ confidence or anxiety during the subsequent HF simulation. Because the order of simulations was determined by the fixed clerkship schedule, we could not manipulate or counterbalance it. Considering that repeated simulation can positively influence affective outcomes [21], future research needs to counterbalance or randomize the order of simulation modalities to clarify the distinct effects of each on students’ emotional and subjective outcomes.

While the overall scores revealed no significant differences between the two simulation methods, the analysis of individual items showed significant differences. The items belonging to Cognitive Control in the “Flow in Education Scale” and Cognitive Domain in the “Perceived Learning Scale” showed a higher score in the VR simulation than in the HF simulation. These results raise the intriguing possibility that VR simulations may be more effective for cognitive learning than HF simulations.

VR simulation is known to be more effective for gaining knowledge than for developing skills [43], with particular strength in enhancing procedural knowledge and skills [44,45]. For example, medical students who trained using a VR simulator demonstrated better procedural knowledge acquisition and retention than those who trained using computerized virtual patient simulations, particularly in managing clinical scenarios such as diabetic ketoacidosis and sepsis [46]. Similarly, VR simulation resulted in greater surgical procedural skills in subdermal drain placement and surgical dissection than video lectures with a VR recording [47]. Integrating the findings of this study with prior research, VR simulation appears to have a great impact on cognitive learning. Prior findings have demonstrated the superiority of VR simulations over conventional teaching methods, such as didactic lectures or videos [47–49]. This study further suggests that VR simulations may have a greater impact than HF simulations on acquiring procedural knowledge and skills.

In summary, this study found that VR simulation did not produce significantly different educational outcomes compared to HF simulation in terms of emotional and subjective experience. Even if the absence of differences is not attributed to sample size or measurement, this study has limitations in generalizability. First, the study was conducted with a single NRP scenario in a pediatric clerkship; further investigation with different topics and other specialties is needed. Second, because it was the second VR simulation in the students’ clerkship, they may not have had sufficient experience with the simulators. The results of the study could be confounded by their lack of skill or the novelty of the VR simulation. In addition, given that the expertise of the learner impacts learning in VR simulations [42,50], a study with various learners, such as junior students or residents, is needed. Finally, as the study focused solely on students’ subjective variables, it did not confirm whether they truly affected their clinical performance. A follow-up study is needed to explore the relationship between subjective variables and objective outcomes such as test scores and clinical skills. Nevertheless, this study is meaningful as it compared VR and HF simulations using standardized scales to investigate their impact on subjective outcomes and highlighted VR’s effect on students’ responses and perceptions during simulation. Simulation-based learning activates both positive and negative emotions [19,51]. As sensory and emotional responses can stimulate cognitive learning (e.g., [52]), understanding emotional responses during simulation is essential for optimizing simulation-based education

VR simulation offers distinct advantages over HF simulation in terms of practical implementation. A prior study found that VR simulation costs less per single-learner session than HF simulation [30]. Zendejas et al. [53] pointed out that HF simulations incur significant costs from the initial purchase of mannequins to the management of infrastructure and simulators. In addition, HF simulation requires a spacious room to set up and control simulators. Compared to this, VR simulation is a more space-efficient alternative. The cost and space efficiency of VR simulation present a significant opportunity for its adoption in schools, making it a highly applicable educational model.

Moreover, VR simulation enables independent and self-directed learning because it operates autonomously, adapts to the student’s performance in real time, and records the entire process for review and feedback [54]. As it does not require the assistance of an operator, students can run and repeat the simulation independently, offering highly accessible and flexible simulation training [55]. Students can effectively enhance their clinical competence, including procedural knowledge and skill proficiency, through repeated self-directed training (e.g., [42]). Iterative simulations boost confidence and reduce anxiety [26,42], and the feedback further improves their learning [56]. Moreover, by using recorded performance data and feedback from the system, students can reflect on their actions and regulate their learning, thus fostering metacognitive skills [55]. Ultimately, VR simulations can facilitate student-centered learning and enable individualized education. Given its cost and space efficiency, and the potential for self-directed repeated practice, VR simulation holds significant promise for widespread application in medical education. Although this study found no significant differences between VR and HF simulations, VR’s cost- and space-efficiency and its support for autonomous learning highlight the need for further research, particularly on affective response and cognitive learning.

## Supporting information

S1 File The complete survey questions. (PDF)

S2 File PlosOne_Datafile. (XLSX)
