# Supplementary material for: Effects of virtual reality simulation on medical students’ emotional and subjective experience compared to high-fidelity simulation in pediatrics clerkship
Source: PLoS One. 2025 Oct 8;20(10):e0323150. doi: 10.1371/journal.pone.0323150 (PMC12507253; doi:10.1371/journal.pone.0323150)
Supplement: S1 File — (PDF) [file pone.0323150.s001.pdf]

The complete survey questions (English)

Variable /Scale	Question items	Not at all	Somewhat	Neutral	Moderately	Very much
Anxiety /STAI (Marteau, Bekker, 1992)	I feel calm.					
	I feel tense.					
	I feel upset.					
	I feel relaxed.					
	I feel content.					
	I feel worried.					

Variable /Scale	Question items	Very anxious	Slightly anxious	Neutral	Moderately confident	Very confident
Confidence /Cato(2013)	Caring for a patient in the simulation room environment contributes to my feeling __:					
	Working with the medical equipment in the simulation room contributes to my feeling__:					
	Distinguishing between what is real and what is simulated (like patient assessment data or operation of equipment) contributes to my feeling__:					
	When working with the mannequin I feel__:					
	Being "on camera" contributes to my feeling__:					
	Caring for a patient with my team contributes to my feeling__:					
	When making a decision about the patient I feel__:					
	Performing in front of my peers contributes to my feeling__:					
	Performing in front of faculty contributes to my feeling__:					

	The possibility of making a mistake contributes to my feeling__:					

**Variable /Scale**	**Question items**	Not at all	Somewhat	Neutral	Moderately	Very much
Engagement / Flow in Education Scale(Heutte et al., 2021)	**Cognitive control**					
	I feel I am able to meet the high demands of the situation.					
	I feel that what I do is under my control.					
	I know what I have to do at every step of the task.					
	**Time transformation**					
	Time seems to flow by in a different way than ever before.					
	I feel like the time is flying very fast.					
	I don't notice the time passing.					
	**Loss of self-consciousness**					
	I didn't care about what the others could think of me.					
	I don't fear the judgment of others.					
	I was not worrying about what the others think about me.					
	**Autotelic experience**					
	I have the feeling of living a moment of excitement.					
	This activity makes me happy.					
	When I talk about this activity, I feel a strong emotion and I want to share it.					

**Variable /Scale**	**Question items**	Strongly Disagree	Disagree	Neutral	Agree	Strongly Agree
Perceived learning / Perceived	**Cognitive**					
	I can organize course material into a logical					

Learning Scale(Rovai et al.,2009)	structure.					
	I cannot produce a course study guide for future students.					
	I can intelligently critique the texts used in this course					
	**Affective**					
	I have changed my attitudes about the course subject matter as a result of this course.					
	I feel more self-reliant as the result of the content learned in this course.					
	I feel that I am a more sophisticated thinker as a result of this course.					
	**Psychomotor**					
	I am able to use physical skills learned in this course outside of class.					
